# Editorial: Efficacy and mechanism of herbal medicines and their functional compounds in preventing and treating cardiovascular diseases and cardiovascular disease risk factors

**DOI:** 10.3389/fphar.2023.1236821

**Published:** 2023-07-03

**Authors:** Tiantian Meng, Yuqing Zhang, Jie Wang, Chen Huei Leo, Zhongfeng Li, Jian Zhang, Kuo Gao, Qingyong He

**Affiliations:** ^1^ Department of Cardiology, Guang’anmen Hospital, China Academy of Chinese Medical Sciences, Beijing, China; ^2^ Department of Health Research Methods, Evidence, and Impact, McMaster University, Hamilton, ON, Canada; ^3^ Department of Science, Mathematics and Technology, Singapore University of Technology and Design, Singapore, Singapore; ^4^ Department of Chemistry, Capital Normal University, Beijing, China; ^5^ Shanghai Key Laboratory of New Drug Design, School of Pharmacy, East China University of Science and Technology, Shanghai, China; ^6^ Beijing University of Chinese Medicine, Beijing, China

**Keywords:** cardiovascular disease, cardiovascular disease risk factors, herbal medicine, efficacy, mechanism

## Introduction

Cardiovascular disease (CVD) ranks among the leading causes of mortality worldwide, posing a substantial global public health challenge (Roth et al., 2020). Prevalent CVDs, such as coronary heart disease (CHD), atherosclerosis (AS), and heart failure (HF), afflict hundreds of millions of individuals across the globe. Prevalent cases of CVD nearly doubled from 271 million in 1990 to 523 million in 2019 (Roth et al., 2020). In addition, risk factors for CVD, including dyslipidemia, diabetes, and hypertension, are recognized as critical contributors to the onset of CVDs. The persistently rising incidence of CVD underscores the limitations of current therapeutic approaches, necessitating the identification of novel alternative treatments.

In recent years, herbal medicines have increasingly demonstrated their potential as alternative therapies for cardiovascular-related diseases (Chen et al., 2022; Lai et al., 2022; Meng et al., 2022). However, given the scarcity of clinical evidence, further investigation is warranted to elucidate the efficacy and underlying mechanisms of herbal medicine in treating CVDs.

The purpose of this Research Topic was to probe the effectiveness and mechanistic basis of herbal medicines in treating CVDs. A total of 15 papers were included in our Research Topic, including 6 original articles and 9 reviews.

## Atherosclerosis

AS is a chronic inflammatory disease of the vasculature, characterized by the formation of fibrous and fatty lesions on arterial walls, with a complex pathogenesis (Tall and Bornfeldt, 2023).

In this Research Topic, a mini review by Guo et al. showed that emodin exerted antiatherogenic effects through its anti-inflammatory effects, regulation of lipid metabolism, and protection of endothelial cells. Furthermore, Liu et al. suggested that ginseng could also delay AS progression by antioxidant activity, modulation of autophagy, and inhibition of apoptosis. Moreover, the influence of gut microbiota on AS has garnered increasing interest in recent years. Herbal medicine demonstrates significant therapeutic effects in modulating gut microbiota and ameliorating AS. Xie et al. demonstrated that ginsenoside Rc (GRc) could play an antiatherogenic role by regulating gut microbiota and fecal metabolites *in vivo*. Li et al. further reviewed the mechanism of gut microbiota in AS, concluding that gut microbiota played an essential role in the occurrence and aggravation of AS by modulating immunity and metabolism, which indicated that vascular mitochondria might be the key medium for gut microflora disorder. Certain herbal medicines and their components, including berberine, resveratrol, allicin, quercetin, and curcumin, could regulate both the gut microbiota and vascular mitochondria to exert therapeutic effects on AS plaques. In addition, AS encompasses various cellular processes and signaling pathways, with the mTOR signaling pathway emerging as a crucial regulatory route. Wu et al. summarized the influence of the mTOR signaling pathway on AS plaques, with a focus on lipid metabolism, autophagy, immune response, and antiaging. Their findings indicated that targeting the inhibition of the mTOR signaling pathway might represent a promising avenue for the treatment of AS. Botanical drugs containing natural compounds, such as quercetin and resveratrol, could modulate autophagy, attenuate immune responses, and delay AS progression by inhibiting the mTOR signaling pathway.

## Hypertension

Hypertension is characterized by a systemic elevation in arterial blood pressure, defined as office systolic blood pressure ≥ 130 mmHg and diastolic blood pressure ≥ 80 mmHg (Tschanz et al., 2020). Despite considerable advancements in pharmacological interventions, the treatment and control of hypertension continue to be suboptimal. In recent years, emerging evidence has highlighted the potential of herbal medicine as an alternative therapeutic approach for hypertension (Lai et al., 2022; Meng et al., 2022).

In this Research Topic, a meta-analysis by Lin et al. demonstrated that Banxia Baizhu Tianma decoction (BXD) combined with conventional therapy (CT) significantly reduced blood pressure compared to CT alone. Additionally, in terms of blood lipids, inflammation, homocysteine, and endothelial function, the combination of BXD and CT was superior to CT alone. Network pharmacology suggested that the drug targets might be ACE, AKT1, NOS3, and PPARG. Guo et al. summarized the regulatory mechanism of emodin on blood pressure and found that emodin played an antihypertensive role by inhibiting deendothelialized aortic vasoconstriction induced by phenylephrine. The combination of emodin and irbesartan was found to alleviate myocardial fibrosis in hypertensive rats.

## Platelet aggregation and thrombus

Platelet aggregation and thrombosis can result in partial or complete occlusion of blood vessels (Li et al., 2022). Although certain antithrombotic agents have demonstrated efficacy, maintaining the balance between embolism prevention and bleeding risk remains challenging (Kwaan and Bennett, 2012).

Qin et al. provided a comprehensive summary of the antithrombotic potential of Astragali Radix and its active metabolites, revealing their ability to protect against vascular endothelial cell damage, decrease coagulation factor levels, and inhibit the fibrinolytic system. Additionally, the combination of Astragali Radix and anti-platelet drugs such as aspirin or clopidogrel could significantly enhance the antiplatelet effect and reduce the risk of bleeding. Liu et al. reviewed the antithrombotic mechanism of ginseng. This review showed that ginsenosides played an antithrombotic role by improving platelet aggregation, suppressing thrombin, and inhibiting fibrin binding with IIb/β3 on platelets. Furthermore, synergistic interactions among various ginsenosides could amplify the overall antithrombotic effect.

## Cornary heart disease

CHD is characterized by coronary artery stenosis or occlusion caused by atherosclerotic lesions. Common manifestations of CHD encompass stable angina pectoris (SAP), unstable angina pectoris (UAP), and myocardial infarction (MI). As clinical evidence accumulates, herbal medicine is emerging as an alternative approach for primary and secondary prevention in patients with CHD (Lv et al., 2022; Ma et al., 2023).

In this Research Topic, a meta-analysis by Wei et al. showed that the combination of Shexiang Baoxin pill and CT is superior to CT alone in reducing cardiovascular adverse events, improving cardiac function, and alleviating angina. In another meta-analysis, Xi et al. evaluated the efficacy of Xueshuantong (lyophilized) injection (XST) for UAP. The results showed that patients received XST combined with CT experienced fewer angina episodes and exhibited reduced levels of cholesterol, triglycerides, whole blood viscosity and plasma viscosity. Tan et al. adopted improved network pharmacology combined with metabolomics to investigate the molecular mechanism of Huoxue Qingre decoction (HXQR) for CHD. It was found that puerarin, kaempferol, luteolin, baicalein, and tanshinone iia might be the predominant active ingredients of HXQR for CHD.

MI arises from acute and sustained ischemia and hypoxia in the coronary artery, leading to subsequent myocardial cell death. Li et al. demonstrated that Suxiao Jiuxin pill (SJP) could relax the coronary artery by the Akt-eNOS-NO pathway and thereby protect against acute MI in rats. A mini review by Guo et al. summarized the therapeutic effect of emodin in MI, suggesting that this compound might prevent myocardial cell damage by inhibiting apoptosis and inflammatory responses.

## Myocardial ischemia-reperfusion injury

Myocardial ischemia‒reperfusion (I/R) injury refers to the damage inflicted on myocardial cells during the reperfusion of coronary arteries. Its pathogenesis is primarily associated with oxidative stress, inflammation, calcium overload, energy metabolism disorder, apoptosis, and ferroptosis (Li et al., 2018; Fan et al., 2021; Wang et al., 2022; Xia et al., 2022). The results of single-aspect treatment are unsatisfactory. Due to its multicomponent and multitarget nature, herbal medicine may provide new insights into myocardial I/R injury.

Li et al. conducted a meta-analysis to assess the clinical and preclinical evidence supporting the efficacy of curcumin in treating myocardial I/R injury. The meta-analysis of clinical studies showed that curcumin could decrease the incidence of cardiac insufficiency and major adverse cardiovascular events. The meta-analysis of preclinical studies showed that curcumin exerted cardioprotective effects mainly by antioxidant, anti-inflammatory, antifibrotic and antiapoptotic effects. Liu et al. showed that ginsenosides could protect the heart from myocardial I/R injury through antioxidant effects, potentially linked to the reduction in intracellular calcium overload and the inhibition of the JNK signaling pathway. Another review by Guo et al. showed that emodin could confer protection against myocardial I/R injury by modulating inflammation, oxidative stress, and apoptosis in MI models.

## Heart failure

HF represents the end stage of various CVDs and constitutes a complex clinical syndrome (Groenewegen et al., 2020). Current standard treatment for HF remains unsatisfactory.

Du et al. conducted a randomized controlled trial, demonstrating that Qishen granule (QSG), when added to standard treatment, improved N-terminal pro-B-type natriuretic peptide levels, six-min walking distance, New York Heart Association class, and quality of life in patients with chronic HF compared to placebo. Lv et al. provided a comprehensive overview of the clinical evidence and mechanism of Yiqi Fumai injection (YQFM) for HF. This study indicated that YQFM could improve heart function, inhibit ventricular remodeling, and enhance quality of life in patients with HF through multiple mechanisms, including anti-inflammation, anti-apoptosis, antioxidation, regulating miRNA expression, improving mitochondrial function, and maintaining the balance of matrix metalloproteinase and tissue inhibitor of metalloproteinase. In an animal experiment, Yu et al. demonstrated that Linggui Zhugan decoction (LGZG) improved myocardial antioxidant capacity and mitochondrial function by activating the SIRT1/AMPK/PGC-1a signaling pathway, thereby enhancing cardiac function in rats with HF. Liu et al. indicated that ginsenosides could improve ventricular remodeling and alleviate failure by scavenging free radicals, improving antioxidant enzyme function and enhancing metabolic capacity. Moreover, ginsenosides were found to exert protective effects against HF resulting from cardiotoxicity. Guo et al. summarized the mechanism of emodin in treating HF, concluding that emodin could potentially delay HF progression by improving mitochondrial energy metabolism, reducing histone deacetylase activity in cardiomyocytes, inhibiting myocardial fibrosis and regulating calcium homeostasis.

## Conclusion

This Research Topic encompasses 15 articles covering a wide range of studies on herbal medicines, including traditional Chinese medicine preparations and their functional compounds for CVDs and relevant risk factors. Table 1 presents the plant names in the traditional Chinese medicine preparations. These findings significantly enhance our comprehension of the therapeutic effects and mechanisms of herbal medicines for cardiovascular-related diseases. In addition, several potential therapeutic targets for CVDs were identified. Some of these studies have also identified several active ingredients in herbal medicines, providing promising candidates for new drug development. In summary, our Research Topic furnishes scientific evidence supporting the efficacy of herbal medicine in the treatment of CVDs and their risk factors, bolstering the prospects of herbal medicine as an alternative therapeutic approach for CVDs.

**TABLE 1:** Plant names in traditional Chinese medicine preparations.

Preparations	Plant names in the preparations	Source
Shexiang Baoxin pill	Moschus berezovskii, M. sifanicus or M. moschiferus (Shexiang), Panax ginseng C.A.Mey. (Renshen), *Bos taurus* domesticus Gmelin (Niuhuang), Cinnamomum cassia Presl (Rougui), Liquidambar orientalis Mill. (Suhexiang), *Bufo gargarizans* Cantor (Chansu), and Borneolum Syntheticum (Bingpian)	Wei et al.
Xueshuantong injection	Panax notoginseng saponins	Xi et al.
Huoxue Qingre decoction	Salvia miltiorrhiza Bunge (Danshen), Panax ginseng C.A.Mey. (Renshen), Coptis chinensis Franch. (Huanglian), Pinellia ternata (Thunb.) Makino (Fabanxia), Paeonia lactiflflora Pall. (Chishao), Panax notoginseng (Burk) F. H. Chen (Sanqi), Pueraria lobata (Willd.) Maesen and S.M. Almeida ex Sanjappa and Predeep (Gegen), and Cistanche deserticola Ma (Roucongrong)	Tan et al.
Suxiao Jiuxin pill	Ligusticum chuanxiong Hort. (Chuanxiong) and Borneolum syntheticum (Bingpian)	Li et al.
Qishen granule	Astragalus mongholicus Bunge (Huangqi), Salvia miltiorrhiza Bunge (Danshen), Aconitum carmichaeli Debeaux (Wutou), Scrophularia ningpoensis Hemsl. (Xuanshen), *Lonicera japonica* Thunb. (Rendong), and Glycyrrhiza glabra L. (Gancao)	Du et al.
Yiqi Fumai injection	Panax ginseng C.A. Mey. (Renshen), Ophiopogon ja ponicus (Thunb.) Ker Gawl (Maidong), and Schisandra chinensis (Turcz.) Baill (Wuweizi)	Lv et al.
Linggui Zhugan decoction	Poria cocos (Schw.) Wolf (Fuling), Neolitsea cassia (L.) Kosterm. (Guizhi), Atractylodes macrocephala Koidz. (Baizhu), and Glycyrrhiza glabra L. (Gaocao)	Yu et al.
Banxia Baizhu Tianma decoction	Pinellia ternata (Thunb.) Makino (Banxia), Atractylodes macrocephala Koidz. (Baizhu), Citrus × aurantium L. (Chenpi), Glycyrrhiza uralensis Fisch. ex DC. (Gancao), Gastrodia elata Blume (Tianma), and Poria cocos (Schw.) Wolf (Fuling)	Lin et al.

## References

[B2] Chen L. Fu G. Hua Q. Zhu H. Y. Deng Y. Wu W. (2022). Efficacy of add-on danhong injection in patients with unstable angina pectoris: A double-blind, randomized, placebo-controlled, multicenter clinical trial. J. Ethnopharmacol. 284, 114794. 10.1016/j.jep.2021.114794 34732357

[B3] Fan Z. Cai L. Wang S. Wang J. Chen B. (2021). Baicalin prevents myocardial ischemia/reperfusion injury through inhibiting ACSL4 mediated ferroptosis. Front. Pharmacol. 12, 628988. 10.3389/fphar.2021.628988 33935719 PMC8079950

[B4] Groenewegen A. Rutten F. H. Mosterd A. Hoes A. W. (2020). Epidemiology of heart failure. Eur. J. Heart Fail. 22, 1342–1356. 10.1002/ejhf.1858 32483830 PMC7540043

[B5] Kwaan H. C. Bennett C. L. (2012). Adverse effects of drugs on hemostasis and thrombosis. Semin. Thromb. Hemost. 38, 755–758. 10.1055/s-0032-1329596 23129499

[B6] Lai X. Dong Z. Wu S. Zhou X. Zhang G. Xiong S. (2022). Efficacy and safety of Chinese herbal medicine compared with losartan for mild essential hypertension: A randomized, multicenter, double-blind, noninferiority trial. Circ. Cardiovasc. Qual. Outcomes 15, e007923. 10.1161/CIRCOUTCOMES.121.007923 35105177

[B7] Li L. Pan C. S. Yan L. Cui Y. C. Liu Y. Y. Mu H. N. (2018). Ginsenoside Rg1 ameliorates rat myocardial ischemia-reperfusion injury by modulating energy metabolism pathways. Front. Physiol. 9, 78. 10.3389/fphys.2018.00078 29467677 PMC5808323

[B8] Li X. Guo T. Feng Q. Bai T. Wu L. Liu Y. (2022). Progress of thrombus formation and research on the structure-activity relationship for antithrombotic drugs. Eur. J. Med. Chem. 228, 114035. 10.1016/j.ejmech.2021.114035 34902735

[B9] Lv J. Liu S. Guo S. Gao J. Song Q. Cui X. (2022). Tongxinluo capsule as supplementation and cardiovascular endpoint events in patients with coronary heart disease: A systematic review and meta-analysis of randomized, double-blind, placebo-controlled trials. J. Ethnopharmacol. 289, 115033. 10.1016/j.jep.2022.115033 35091010

[B10] Ma X. Wang Q. Liu C. Liu J. Luo G. He L. (2023). Regulation of phospholipid peroxidation signaling by a traditional Chinese medicine formula for coronary heart disease. Phytomedicine 114, 154749. 10.1016/j.phymed.2023.154749 36931097

[B11] Meng T. Wang P. Xie X. Li T. Kong L. Xu Y. (2022). Efficacy and safety of songling xuemaikang capsule for essential hypertension: A systematic review and meta-analysis of randomized controlled trials. Phytomedicine 107, 154459. 10.1016/j.phymed.2022.154459 36183476

[B12] Roth G. A. Mensah G. A. Johnson C. O. Addolorato G. Ammirati E. Baddour L. M. (2020). Global burden of cardiovascular diseases and risk factors, 1990-2019: Update from the GBD 2019 study. J. Am. Coll. Cardiol. 76, 2982–3021. 10.1016/j.jacc.2020.11.010 33309175 PMC7755038

[B13] Tall A. R. Bornfeldt K. E. (2023). Inflammasomes and atherosclerosis: A mixed picture. Circ. Res. 132, 1505–1520. 10.1161/CIRCRESAHA.123.321637 37228237 PMC10213995

[B14] Tschanz C. M. P. Cushman W. C. Harrell C. T. E. Berlowitz D. R. Sall J. L. (2020). Synopsis of the 2020 U.S. Department of veterans affairs/U.S. Department of defense clinical practice guideline: The Diagnosis and management of hypertension in the primary care setting. Ann. Intern. Med. 173, 904–913. 10.7326/M20-3798 32866417

[B15] Wang R. Wang M. Liu B. Xu H. Ye J. Sun X. (2022). Calenduloside E protects against myocardial ischemia-reperfusion injury induced calcium overload by enhancing autophagy and inhibiting L-type Ca(2+) channels through BAG3. Biomed. Pharmacother. 145, 112432. 10.1016/j.biopha.2021.112432 34798472

[B16] Xia B. Li Q. Wu J. Yuan X. Wang F. Lu X. (2022). Sinomenine confers protection against myocardial ischemia reperfusion injury by preventing oxidative stress, cellular apoptosis, and inflammation. Front. Pharmacol. 13, 922484. 10.3389/fphar.2022.922484 35837272 PMC9274168

